# AI-based satellite survey offers independent assessment of migratory wildebeest numbers in the Serengeti

**DOI:** 10.1093/pnasnexus/pgaf264

**Published:** 2025-09-09

**Authors:** Isla Duporge, Zijing Wu, Zeyu Xu, Peng Gong, Daniel Rubenstein, David W Macdonald, Anthony R E Sinclair, Simon Levin, Stephen J Lee, Tiejun Wang

**Affiliations:** Department of Ecology and Evolutionary Biology, Princeton University, 11 Ivy Lane, Princeton, NJ 08540, USA; Wildlife Conservation Research Unit, Department of Biology, University of Oxford, Abingdon Road, Oxfordshire OX13 5QL, United Kingdom; Department of Geography, The University of Hong Kong, Hong Kong, SAR 999077, China; ITC, Faculty Geo-Information Science and Earth Observation, Hallenweg 8, 7522 NH Enschede, The Netherlands; Department of Geography, The University of Hong Kong, Hong Kong, SAR 999077, China; Institute for Climate and Carbon Neutrality, The University of Hong Kong, Hong Kong, SAR 999077, China; Department of Ecology and Evolutionary Biology, Princeton University, 11 Ivy Lane, Princeton, NJ 08540, USA; Wildlife Conservation Research Unit, Department of Biology, University of Oxford, Abingdon Road, Oxfordshire OX13 5QL, United Kingdomc; Biodiversity Research Centre, University of British Columbia, 2212 Main Mall, Vancouver, BC, Canada V6T 1Z4; Department of Ecology and Evolutionary Biology, Princeton University, 11 Ivy Lane, Princeton, NJ 08540, USA; DEVCOM Army Research Laboratory, 800 Park Offices Dr, Durham, NC 27703, USA; ITC, Faculty Geo-Information Science and Earth Observation, Hallenweg 8, 7522 NH Enschede, The Netherlands; School of Geospatial Engineering and Science, Sun Yat-sen University, Zhuhai, Guangdong Province 519082, China

## Abstract

The Great Wildebeest Migrationin the Serengeti-Mara ecosystem is a globally iconic wildlife phenomenon that supports the health and biodiversity of the region by supporting predator populations, regulating herbivore densities, and driving nutrient cycling. This study presents the first AI-powered satellite survey, using two deep learning-based models (U-Net and YOLOv8) to detect and count wildebeest over more than 4,000 km² across two consecutive years in August 2022 and 2023 with F1 scores reaching 0.830 (Precision: 0.832, Recall: 0.838). The satellite-based results show fewer than 600,000 individuals—approximately half the widely cited estimate of 1.3 million wildebeest, which has remained largely unchanged since the 1970s. While some variation may arise from differences in spatial and temporal coverage between survey methods, the satellite approach employs rigorously validated AI models with demonstrated accuracy. Rather than undermining previous methods, this discrepancy underscores the importance of using independent and complementary monitoring tools to refine population estimates and improve our understanding of wildebeest movement dynamics.

The Great Wildebeest Migration in the Serengeti-Mara ecosystem is integral to ecosystem plasticity and health, vegetation composition, structure, and a complex web of trophic interactions. It is particularly crucial for predator survival, sustaining one of the highest densities of large carnivores in the world, such as lions (*Panthera leo*), spotted hyenas (*Crocuta crocuta*), and Nile crocodiles (*Crocodylus niloticus*). As one of the largest terrestrial mammal migrations on Earth, this phenomenon also attracts high numbers of international tourists, providing an important source of revenue for Kenya and Tanzania.

It has been widely cited, using aerial point sampling surveys, that the population of migratory wildebeest (*Connochaetes taurinus*) in the Serengeti has grown from an estimated 250,000 individuals in the 1950s, before livestock were vaccinated against rinderpest to more than 1.3 million today, with some drought-related oscillations since 1977 (1). These estimates stem from photographic aerial point sampling surveys. The most recent survey in 2023 was conducted by fixed-wing aircraft flying a straight-line distance of 2,332 km, 2.5 km apart, during which wildebeest were counted over three days in the southern part of the Serengeti in Tanzania after the wildebeest had calved (2). The Jolly II method (3) was then used to multiply the average density within each image to extrapolate the count to a survey area of 4,782 km^2^. Manned aircraft surveys of wildlife have established the most comprehensive ecological datasets to date and have been instrumental in conservation efforts across the African continent. However, the need for statistical extrapolation to unsurveyed areas introduces the potential for error (4).

Satellite surveys minimize double counting as much larger areas can be covered at one time. Additionally, this method avoids disturbing wildlife and removes risks to human safety. This is relevant given that aircraft crashes are a leading cause of death among wildlife biologists (5). While scheduling satellite image acquisition at precise times is challenging due to cloud cover and fixed orbital paths, the growing number of satellite constellations with improved spatial and temporal resolution is reducing these limitations.

The use of satellite imagery to detect wildlife has expanded over the past decade for species that have a large enough body size or exhibit sufficient spectral contrast for detection (6). While unmanned aerial surveys are promising survey tools, current battery capabilities significantly limit coverage (7). Satellite detection is highly suited to monitoring wide-ranging species as individuals may move between transects during aerial surveys, which are often conducted days or weeks apart due to weather constraints. As the highest commercially available satellite resolution is currently limited to 30 cm per pixel, detecting smaller species remains challenging. Beyond image costs, the primary challenge for wildlife biologists using satellite surveying is the sheer volume of imagery, making manual screening of vast areas impractical, necessitating computational expertise to utilize deep-learning object-detection models. Automation expedites counts and improves detection accuracy, and in some applications AI classification exceeds human performance in both consistency and accuracy (8). While the advantage of using deep learning-based satellite surveying has been demonstrated in previous studies (9) its application for estimating population abundance remains limited. Notable examples include satellite surveys of penguins (Spheniscidae) that revealed previously unknown colonies by detecting guano stain (10) and population assessments of Weddell seals (*Leptonychotes weddellii*) (11) and albatrosses (*Diomedeidae*) (12).

This study is the first demonstration of the ability to conduct an abundance estimate of a terrestrial mammal from satellite illustrating the feasibility of estimating terrestrial mammal populations using satellite imagery and machine learning, providing a scalable approach for wildlife monitoring which can be expanded to other wildlife populations. To conduct the count a method that was previously developed, rigorously tested, and validated to count migratory wildebeest is applied (13) across the northern Serengeti National Park in Tanzania and southwestern Kenya, encompassing the Masai Mara National Reserve over two consecutive years in 2022 and 2023. These areas form a continuous ecosystem and are part of the expansive Serengeti-Mara ecosystem. The satellite coverage spans an area of 4,458 km^2^ in 2022 (Fig. 1) and 4,047 km^2^ in 2023 (Fig. 2), with image resolutions ranging from 33 to 60 cm, capturing each wildebeest as a 6- to 12-pixel representation. To accurately identify and count wildebeest, we utilize two advanced deep-learning models: U-Net, a pixel-based segmentation model, and YOLOv8, an object-based detection model, and frameworks tailored for this specific task have been developed. Applying both models allows us to compare their efficacy in population estimation within this large-scale wildlife survey.

**Fig. 1.**
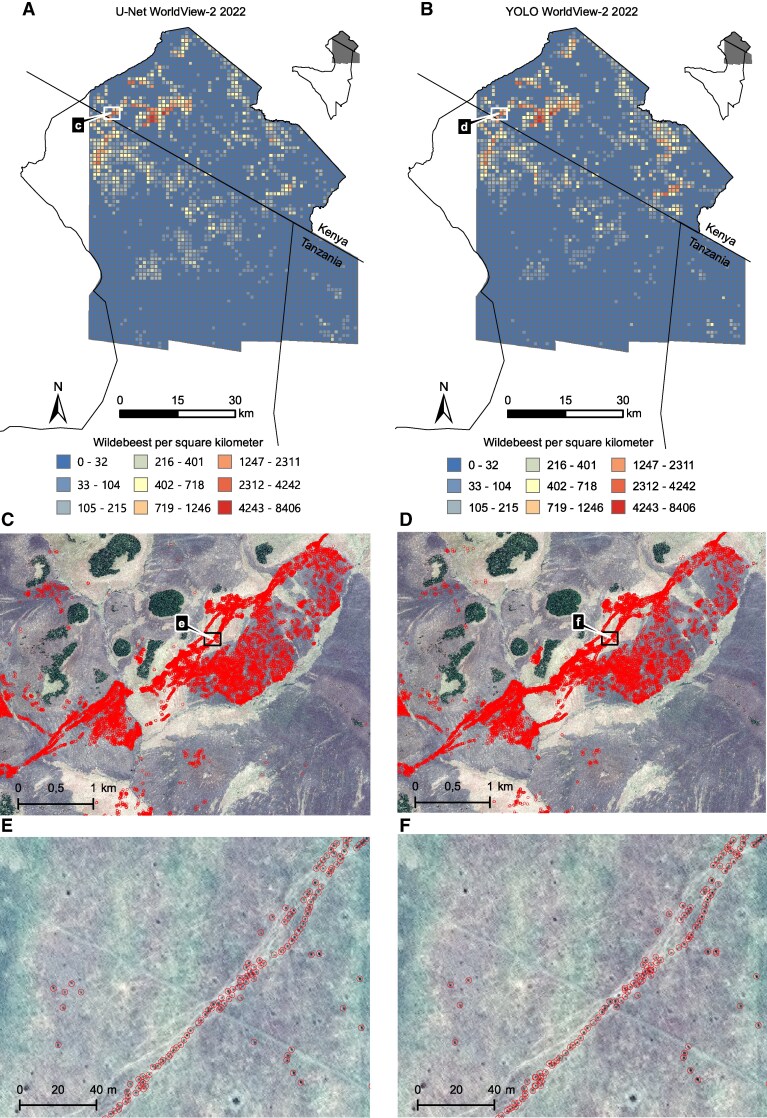
Wildebeest distribution from WorldView-2 imagery (2022 Aug 6). U-Net results shown on the left (A, C, E); YOLOv8 results on the right (B, D, F). A and B) Density maps (wildebeest/km²); C–F) The circles indicate detections at increasing zoom. Imagery © 2022 Maxar Technologies.

**Fig. 2.**
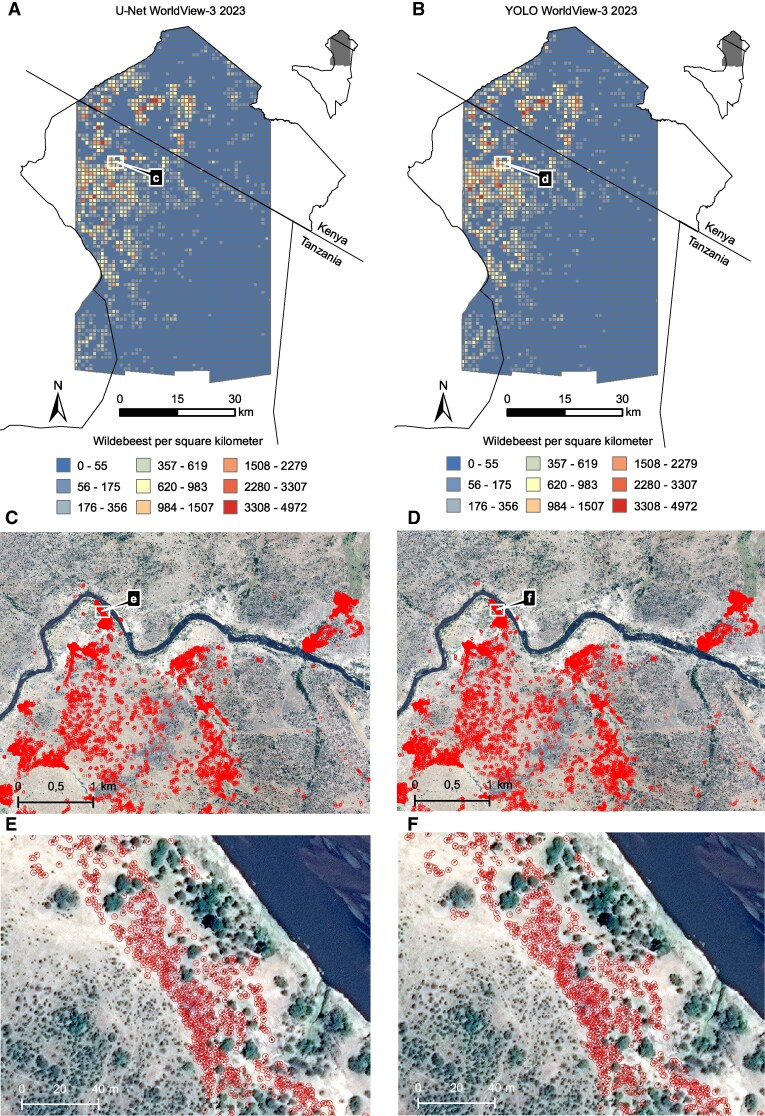
Wildebeest distribution from WorldView-3 imagery (2023 Aug 28). U-Net results shown on the left (A, C, E); YOLOv8 results on the right (B, D, F). A and B) Density maps (wildebeest/km^2^); C–F) The circles indicate detections at increasing zoom. Imagery © 2023 Maxar Technologies.

The models yielded comparable results, with U-Net achieving an F1-score (the harmonic mean of precision and recall) of 0.729 (Precision: 0.722, Recall: 0.739) in 2022 and 0.830 (Precision: 0.832, Recall: 0.838) in 2023, while YOLOv8 achieved F1-scores of 0.760 (Precision: 0.739, Recall: 0.781) in 2022 and 0.771 (Precision: 0.743, Recall: 0.802) in 2023. Estimated counts from U-Net in 2023 reached 502,917, with YOLOv8 estimating 533,137; for 2022, U-Net estimated 324,202 and YOLOv8 337,926. The distributions of detected wildebeest for the 2 years are presented in Figs. 1 and 2.

The discrepancy between the population estimates from satellite and aerial counts invites careful consideration of where the “missing” wildebeest might be. While it is possible that some individuals are missed in satellite imagery due to canopy cover, it seems unlikely that such a large number—on the order of half a million—would be entirely concealed. Although some wildebeest may have been dispersed in areas like the western corridor, available data from other sources indicate that the majority of the migratory herds are within the satellite's surveyed region at the time of image acquisition. Notably, the Great Migration remains a synchronized movement of mixed herds rather than a sex-segregated migration, even during the dry season. While minor spatial differentiation may occur at localized scales, males and females fundamentally follow the same migratory routes. Additionally, crowd-sourced sightings data also corroborate the presence of wildebeest in the imaged area. Although crowd-sourced data contain some observational biases, it remains a valuable supplementary source—especially in regions where systematic monitoring is sparse.

The count generated from this survey may even be considered an overestimate, as it includes detections of zebras and potentially elands, which often travel with wildebeest herds but have not been differentiated from wildebeest. These species cannot be visually distinguished at the current satellite spatial resolution—future research incorporating validated ground-truth labels could use spectral signatures to enable differentiation.

The migratory wildebeest population could be lower than previous population estimates for the same population in the south of the ecosystem due to a combination of ecological and anthropogenic factors. Habitat fragmentation, driven by agricultural expansion, infrastructure development, and fencing, has reduced the available space for wildebeest migration routes (14), while climate change is altering seasonal rainfall patterns, affecting the timing and quality of grazing resources essential for their migration (15). Additionally, predation rates on wildebeest calves and poaching levels may have further contributed to population declines (16).

An alternative explanation is an incomplete understanding of migration patterns. Habitat variability under current climatic conditions may be causing mega herds to fragment across larger areas. Behavioral plasticity in the selection of migration routes may be causing wildebeest to disperse more thinly across a much broader territory than previously documented, with wildebeest no longer crossing the Mara River into Kenya—the northern part of their traditional range. Additionally, unusual dry-season rains and postburn regrowth in the western and central ecosystem can alter wildebeest movements. To account for the full differential in population estimates between aerial surveys and satellite surveys, we would need to use the highest estimate of 533,137 individuals over 4,047 km^2^ in 2023 from U-Net and extrapolate this, assuming similar densities over 10,000 km^2^, to reach an estimate of close to 1,300,000. If true, this is a profound difference in our understanding of wildebeest migration dynamics compared to that currently established. To resolve the discrepancy between the population numbers, it is necessary to calibrate a satellite survey against a manned aircraft survey at the same time to quantify the relative bias errors.

We compare our results to manned aerial surveys not because aerial surveys represent a definitive ground truth, but as the most established reference currently available—underscoring the need for methodological pluralism. Satellite-based wildlife monitoring provides consistent and scalable data, making it well-suited to augment existing wildlife monitoring frameworks.

## Supplementary Material

pgaf264_Supplementary_Data

## Data Availability

Methods are contained in Supplementary material and code to reproduce the results is available at github.com/sat-wildlife/wildebeest. The satellite imagery cannot be shared as it is propriety and belongs to Maxar Technologies, but instructions to access the imagery are provided in the Supplementary material.
