# Correction to: AI-based satellite survey offers independent assessment of migratory wildebeest numbers in the Serengeti

**DOI:** 10.1093/pnasnexus/pgaf382

**Published:** 2025-12-17

This is a correction to: Isla Duporge, Zijing Wu, Zeyu Xu, Peng Gong, Daniel Rubenstein, David W Macdonald, Anthony R E Sinclair, Simon Levin, Stephen J Lee, Tiejun Wang, AI-based satellite survey offers independent assessment of migratory wildebeest numbers in the Serengeti, *PNAS Nexus*, Volume 4, Issue 9, September 2025, pgaf264, https://doi.org/10.1093/pnasnexus/pgaf264

In the originally published version of the manuscript, the authors note the following corrections.

In the interests of accuracy, the following sentences have been deleted: “Global Positioning System (GPS) tracking data from both years supports this conclusion. Fixes taken 1 h before image acquisition show that 28 collared females in 2023 and 20 in 2022 were located within the surveyed region, with only two individuals outside.”

The authors wish to clarify that original phrasing may lead to the misunderstanding that the wildebeest count represented a total population estimate. The count obtained from satellite imagery represents only those animals visible within the surveyed area at the time of image acquisition. Because the total distribution of the herd extends beyond this area, extrapolation to the entire population is not justified, and no inference can be made about the accuracy of previous estimates. The study demonstrates that satellite imagery provides a valuable complementary tool that should be calibrated against conventional survey methods. This clarification does not alter the study’s main conclusion that AI-based satellite surveys provide an independent and complementary means of assessing wildlife populations.

