# Supplementary material for: AI-based satellite survey offers independent assessment of migratory wildebeest numbers in the Serengeti
Source: PNAS Nexus. 2025 Sep 9;4(9):pgaf264. doi: 10.1093/pnasnexus/pgaf264 (PMC12418379; doi:10.1093/pnasnexus/pgaf264)
Supplement: pgaf264_Supplementary_Data [file pgaf264_supplementary_data.pdf]

AI-based satellite survey offers independent assessment of migratory wildebeest numbers in the Serengeti

Isla Duporge[1]*†, Zijing Wu[2]†, Zeyu Xu[3]†, Peng Gong[2,4], Daniel Rubenstein[1], David W. Macdonald[5], Anthony R. E. Sinclair[6], Simon Levin[1], Stephen Lee[7], Tiejun Wang[3]*

* Isla Duporge and Tiejun Wang. Email: Isla.duporge@princeton.edu and t.wang@utwente.nl

SI Appendix

Methods

Satellite image acquisition

The spatial and temporal migration patterns of wildebeest in the Serengeti-Mara ecosystem have been extensively documented in previous studies[1–5] and through real-time tracking platforms such as Serengeti Tracker[6], HerdTracker[7], and Wildebeest Sightings. These sources consistently indicate that by August, the majority of migratory wildebeest move into the Masai Mara National Reserve and the northernmost section of Serengeti National Park. We tasked satellite imagery on August 6, 2022, and August 28, 2023, in the Serengeti-Mara ecosystem through Maxar Technologies and obtained eight multispectral images captured by two satellite sensors: WorldView-2 and WorldView-3. Images acquired in both years can be viewed at the following links: For full image ID information please see – https://xpress.maxar.com/ and use the image IDs below.

2023 Image IDs	Time of Acquisition	% off-nadir
1040010089C37300	08:12:17 UTC	27%
1040010089383500	08:11:42 UTC	14%
104001008AB0C300	08:11:21 UTC	22%
104001008BC29100	08:11:01 UTC	25%

2022 Image IDs	Time of Acquisition	% off-nadir
10300100D876E700	08:24:59 UTC	28%
10300100D78E8000	08:24:45 UTC	28%
10300100D8098B00	08:24:30 UTC	27%
10300100D834AA00	08:24:15 UTC	29%

Both image sets were captured in a single satellite pass. Our tasking request for imagery was for as large an image as it is possible to capture with these sensors and the resulting images cover four adjacent satellite swaths in both years. The images mainly cover the Masai Mara National Reserve and the northernmost section of the Serengeti National Park. The images cover 4,458 km² in 2022 and 4,047 km² in 2023. The spatial resolution varies from 33–38 cm in 2023 and 58–60 cm in 2022. The images were delivered as pan-sharpened products, and the pre-processed satellite images have four bands: Red, Green, Blue, and Near-Infrared. Only Red, Green, and Blue bands were used in the wildebeest detection model, as including Near-Infrared did not offer any additional benefit.

Training dataset preparation

We adopted the wildebeest annotation data from a previous study[8] to train this model. The annotations were digitized on the satellite imagery collected from sensors GeoEye-1, WorldView-2, and WorldView-3 in 2009, 2010, 2013, 2015, 2018, and 2020. This dataset consists of 3,797 image patches, each sized 336 by 336 pixels. Each wildebeest was labeled as a point centered at the group of wildebeest pixels. The final labels for the wildebeest were established through majority consensus by four experienced image interpreters, who visually examined the same satellite image. Additionally, to differentiate wildebeest from other similar spectral objects—such as small bushes or termite mound shadows—we cross-referenced these interpretations against a reference satellite image taken in a different year. The reference imagery provided a stable baseline for comparison, as it included static elements, like bushes and mound shadows, that helped clarify the identification of each wildebeest.

To utilize this wildebeest training dataset on the new satellite imagery in 2022 and 2023, we employed a histogram matching method to adapt the original training dataset to the data distribution of the new dataset. Preliminary experiments showed that a training dataset with more diverse variations in the data distribution yielded better performance. Therefore, we applied histogram matching to the original dataset using the image strips with distinct spectral differences and combined all the versions for final training. Specifically, in 2022, the spectral differences between the two east and two west stripes were significant, so separate training data was created for each side. For each side, 100 random 336 by 336 pixel sub-images were extracted to perform histogram matching on the original samples, resulting in two new groups of samples. Additionally, 100 sub-images were selected from the mosaic image of all four stripes for histogram matching,

resulting in a third new group of samples. These three groups of new samples and the original samples were used simultaneously for training the model in 2022.

For the 2023 training, 100 sub-images were randomly selected from each stripe to perform histogram matching on the original samples, generating a new group of samples. Another 100 sub-images were extracted from the mosaic image of the stripes, and histogram matching was applied, generating another new group of samples. These two groups of new samples, along with the original samples, were used simultaneously for training.

Training the U-Net-based ensemble model for wildebeest detection

The U-Net architecture of convolutional neural networks has proved to be efficient for image segmentation tasks and previous study showed that it can be used to segment wildebeest pixels from submeter-resolution satellite imagery[8]. Therefore, the same strategy was used as this study and we applied a U-Net-based ensemble model to train the wildebeest detection model. The wildebeest annotations were converted to segmentation masks by expanding the annotation point to a 3 by 3-pixel segment. Data augmentation was adopted while training to improve the generalization ability of the model. We used a 5-fold ensemble strategy to improve the robustness of the model: the training dataset was divided into 5 folds, out of which 4 folds were used to train the model, and the remaining fold was used for validation. In total, 5 models were trained and validated with varied fold combinations. The 5 models were then applied to satellite imagery, and the predicted probabilities were averaged to get the final ensemble results. We then converted the probability maps into binary segmentation masks using a threshold of 0.5, and then extracted the wildebeest centroid points from the segments using K-means clustering method. The cluster size was determined according to the image resolution and the corresponding wildebeest size. In year 2022, the image resolution is rather coarse (58-60 cm) and the cluster size was set as 9 pixels. While in year 2023, the image resolution is 33-38 cm, and we set the cluster size as 16 pixels.

Training the YOLOv8 object detector model for wildebeest detection

The YOLO series is a well-regarded collection of object detection models with high accuracy, robustness, and proven effectiveness in animal detection[9],[22]. YOLOv8x was chosen for its superior detection performance[23]. We expanded the original point samples into 4×4 pixel boxes

for YOLO training. The model randomly applied data augmentation techniques, including vertical flips, horizontal flips, mosaic transformations, and scaling during training to enhance its performance. We designed a mechanism to automatically adjust the overlap ratio based on object density to address missed detections of small objects. When predicting across the entire region, the model automatically adjusted the overlap ratio based on object density. Densely populated areas, defined as windows with over 30 detected objects, used an 80% overlap ratio, while less dense areas maintained 50%. Furthermore, to eliminate duplicate detections caused by overlapping regions and reduce false positives, we referred to the original image's spatial resolution to remove redundant detection points within each pixel, keeping only the point with the highest confidence. The final confidence threshold was determined based on the maximum F1-score, with the threshold set to 0.13 for 2022 and 0.20 for 2023.

Model evaluation

To evaluate model performance, we selected 2,000 sampling plots from each year's imagery separately using a stratified proportionate random sampling method. Each sampling plot is a 200m × 200m grid cell. The strata of the test dataset were determined according to the wildebeest density in all the grids to capture the spatially imbalanced distribution of wildebeest. We applied one version of the model to the satellite imagery dataset and detected and counted the wildebeest in all the grids, and then divided them into four categories (low density, medium density, high density and very high density) based on the mean and standard deviations of the wildebeest count in the grids. 2,000 sampling plots were randomly selected from the four categories proportionally. Then we annotated the wildebeest within the 2,000 test sampling plots using the annotation protocol as described above in the training dataset preparation step. The same procedure was repeated for year 2022 and 2023 and the performance was evaluated separately.

The annotations in the test sampling plots were then compared against the model predictions to calculate the accuracy. To account for slight variations between the wildebeest centroids identified by the model (from U-Net segments or YOLOv8 bounding boxes) and human interpretations, we applied a search radius of 2 or 3 pixels to adjust for spatial shifts. In 2022, a prediction was deemed correct if it fell within a 2-pixel radius of the annotated point. For 2023, the search radius was increased to 3 pixels due to the higher spatial resolution of the data. A correct prediction is counted as one True Positive (TP); if no point matches the annotated point,

it will be counted as a False Negative (FN). All the remaining prediction points are counted as False Positives (FP). We calculated the Precision, Recall, and the F1-score based on the predictions. Precision was calculated as the ratio between all TPs and all detected positives (the sum of TPs and FPs). Recall was calculated as the ratio between all TPs and all true positives (the sum of TPs and FNs). They measure the detection ability of the model in different aspects. The F1-score is calculated as the harmonic mean of Precision and Recall.

On the 2022 dataset, U-Net achieved F1-scores of 0.851, 0.862, 0.732, and 0.596 for very high, high, medium, and low wildebeest densities, respectively, with an overall F1-score of 0.729. In comparison, YOLOv8 yielded F1-scores of 0.815, 0.865, 0.733, and 0.686 across the same density levels, and an overall F1-score of 0.760. For the 2023 dataset, U-Net showed improved performance with F1-scores of 0.928, 0.900, 0.862, and 0.718 across very high, high, medium, and low densities, respectively, and an overall F1-score of 0.830. YOLOv8 also improved, achieving F1-scores of 0.892, 0.878, 0.813, and 0.619 across the same density categories, with an overall F1-score of 0.771.

Wildebeest observation data

To validate our satellite data analyses, we incorporated two additional datasets provided by local organizations. HerdTracker (https://www.discoverafrica.com/herdtracker/) and the Grumeti Fund (https://grumetifund.org/) supplied sighting data of migratory wildebeest during our survey periods in 2022 and 2023. HerdTracker maintains crowd-sourced records, including georeferenced photos and videos submitted by tourists and local operators, primarily documenting wildebeest crossings at the Mara River. Coordinates from these records for 2022 and 2023 were reviewed to verify that the majority of wildebeest herds were within the surveyed area at the time of satellite tasking. The Grumeti Fund provided observational data collected by field staff and scouts stationed at observation posts, with sightings relayed to an operations room and systematically recorded in EarthRanger (https://www.earthranger.com/). Using these supplementary datasets allowed for an independent verification of the timing and location of wildebeest movements, enhancing the reliability of our analyses.

SI References

1. Pennycuick, L. Movements of the migratory wildebeest population in the Serengeti area between 1960 and 1973. *Afr. J. Ecol.* **13**, 65–87 (1975).

2. Boone, R. B., Thirgood, S. J. & Hopcraft, J. G. C. Serengeti wildebeest migratory patterns modeled from rainfall and new vegetation growth. *Ecology* **87**, 1987–1994 (2006).
3. Torney, C. J., Hopcraft, J. G. C., Morrison, T. A., Couzin, I. D. & Levin, S. A. From single steps to mass migration: the problem of scale in the movement ecology of the Serengeti wildebeest. *Philos. Trans. R. Soc. Lond. B Biol. Sci.* **373**, (2018).
4. Larsen, F. *et al.* Wildebeest migration drives tourism demand in the Serengeti. *Biol. Conserv.* **248**, 108688 (2020).
5. Tracking the great Serengeti wildebeest migration. *National Geographic*.
6. Sightings, W. Wildebeest Sightings - Wildebeest Migration Tracker Map 2025. *Wildebeest Sightings* https://wildebeestsightings.com/ (2024).
7. HerdTracker - Wildebeest Migration Updates. *Discover Africa* https://www.discoverafrica.com/herdtracker/.
8. Wu, Z. *et al.* Deep learning enables satellite-based monitoring of large populations of terrestrial mammals across heterogeneous landscape. *Nat. Commun.* **14**, 3072 (2023).
9. Xu, Z., Wang, T., Skidmore, A. K. & Lamprey, R. A review of deep learning techniques for detecting animals in aerial and satellite images. *Int. J. Appl. Earth Obs. Geoinf.* **128**, 103732 (2024).
